# Early menarche: A systematic review of its effect on sexual and reproductive health in low- and middle-income countries

**DOI:** 10.1371/journal.pone.0178884

**Published:** 2017-06-07

**Authors:** Mobolaji Ibitoye, Cecilia Choi, Hina Tai, Grace Lee, Marni Sommer

**Affiliations:** 1 Department of Sociomedical Sciences, Columbia University Mailman School of Public Health, New York, New York, United States of America; 2 Heilbrunn Department of Population & Family Health Columbia University Mailman School of Public Health, New York, New York, United States of America; London School of Hygiene and Tropical Medicine, UNITED KINGDOM

## Abstract

**Background:**

Adolescent girls aged 15–19 bear a disproportionate burden of negative sexual and reproductive health outcomes in low- and middle-income countries. Research from several high-income countries suggests that early age at menarche is an important determinant of sexual and reproductive health. We conducted this systematic review to better understand whether and how early menarche is associated with various negative sexual and reproductive health outcomes in low- and middle-income countries and the implications of such associations.

**Methods:**

We systematically searched eight health and social sciences databases for peer-reviewed literature on menarche and sexual and reproductive health in low- and middle-income countries. Two reviewers independently assessed all studies for inclusion, overall quality and risk of bias, and performed data extraction on all included studies.

**Results:**

Twenty-four articles met all inclusion criteria–nine of moderate quality and fifteen with several methodological weaknesses. Our review of the minimal existing literature showed that early menarche is associated with early sexual initiation, early pregnancy and some sexually transmitted infections in low- and middle-income countries, similar to what has been observed in high-income countries. Early menarche is also associated with early marriage–an association that may have particularly important implications for countries with high child marriage rates.

**Conclusions:**

Early age at menarche may be an important factor affecting the sexual and reproductive health of adolescent girls and young women in low- and middle-income countries. More research is needed to confirm the existence of the identified associations across different settings and to better understand the process through which early menarche and other markers of early pubertal development may contribute to the increased vulnerability of girls to negative sexual and reproductive health outcomes in low- and middle-income countries. Given the association of early menarche with early marriage, ongoing efforts to reduce child marriage may benefit from targeting efforts to early maturing girls.

## Introduction

There are 1.8 billion adolescents in the world today, the largest generation in history [1]. Globally, this population is highly vulnerable to negative sexual and reproductive health outcomes such as sexually transmitted infections (STIs) including HIV, unintended pregnancy, and the complications that come with early childbirth [2]. This burden falls disproportionately on adolescent girls [3]. In 2013, two-thirds of all new HIV infections among adolescents between the ages of 15–19 occurred in girls [4]. The disparities are even starker in sub-Saharan Africa, where in some countries, girls aged 15–19 are five times more likely to be infected with HIV than boys their age [4]. Furthermore, the consequences of unintended pregnancy and the risks associated with childbirth fall almost entirely on women and girls [5]. Complications from pregnancy and childbirth including maternal hemorrhage and sepsis, and unsafe abortion are among the leading causes of death among adolescent girls and young women aged 10–24 in low- and middle-income countries [6]. Due to their underdeveloped reproductive systems, pregnant adolescents have increased risk from life-threatening complications including obstructed labor and resultant obstetric fistulae [7–9]. They also have a greater risk of eclampsia and pre-eclampsia [7, 10]. Adolescent girls aged 15–19 years are twice as likely as women in their 20s to die during childbirth; girls under the age of 15 have a five times greater maternal mortality risk [11]. In addition, pregnancy in adolescence is associated with increased risk of HIV infection [12].

Menarche, the onset of menstruation, is an often overlooked indicator in public health [13]. Yet, it is a key developmental marker of a girl’s healthy transition from childhood into young adulthood, and an important clinical indicator of girls’ physical, nutritional, and reproductive health [13]. Menarche marks the beginning of a girl’s reproductive life, and has important implications for adolescent sexual and reproductive health outcomes [13]. A substantial body of evidence from high-income countries suggests that early menarche–generally defined as menarche before age 12–increases adolescent girls’ vulnerability to negative sexual and reproductive health outcomes including early pregnancy and childbearing, STIs, early sexual initiation, and sexual violence [14–22]. For example, data from a birth cohort in New Zealand showed that girls who reached menarche at ages 10–11 years were significantly more likely to report being pregnant by age 18 compared to those with a later age at menarche [17]. Specifically, 11% of girls with early menarche reported being pregnant by age 18, compared to 9% and 1.7% among those with menarche at ages 12–13 and 14–15, respectively. In the same birth cohort, 19.2% of those with early menarche reported having an STI by age 18, compared to 8.8% and 6.9% among those with menarche at ages 12–13 and 14–15, respectively [17]. In a study among young women in the United States, age at menarche was directly and positively correlated to age at first sex, which in turn was directly and positively correlated to age at first pregnancy [14]. However, little is known about whether early menarche is also associated with these outcomes in low- and middle-income countries.

Although it is likely that the links between early menarche and sexual and reproductive health in low- and middle-income countries are similar to those found in high-income countries, this might not necessarily be the case due to differences in sociocultural factors related to menarche and sexual and reproductive health. For example, in many low- and middle-income countries, menarche has traditionally served as a cultural rite of passage and marker of adulthood, positioning a girl as ready for marriage; this is not the case in most high-income countries [13]. Thus, early menarche is more likely to be associated with early marriage in low- and middle-income countries. Furthermore, in many low- and middle-income countries, girls’ mobility and social interactions are often restricted once they reach puberty, limiting their opportunities to engage in pre-marital sexual activity [23]. Such restrictions could minimize the correlation between early menarche and early sexual initiation or risky sexual behavior in low- and middle-income countries. Furthermore, differences in contraception access and use between low-, middle- and high-income countries may contribute to differences in the effect of early menarche on early pregnancy and childbirth across the different settings. Early menarche is likely to have a stronger correlation with adolescent pregnancy and childbirth rates for girls in low-income settings, where access to contraceptives is more limited. In addition, the factors that contribute to early menarche, which could in turn affect related sexual and reproductive health outcomes, may differ. For example, recent declines in the age at menarche in low- and middle-income countries have been attributed to improved socioeconomic, health and nutritional status [24, 25]. On the other hand, in high-income countries, earlier age at menarche has been attributed to markers of lower socioeconomic conditions such as family instability, residential instability, and stressful early life conditions [25–28].

Secular trends from several low- and middle-income countries show decreases in age at menarche over the past few decades [29–34]. This may be increasing girls’ vulnerability [35] given the high burden of negative sexual and reproductive health experienced by adolescent girls and young women in such contexts. There is an urgent need to better understand the effect of early menarche on these outcomes, and the process by which it may increase girls’ vulnerability.

To better understand the association between early menarche and sexual and reproductive health outcomes in low- and middle-income countries and identify gaps in the existing evidence, we conducted a systematic review focused on the following questions: (1) Is early menarche associated with negative sexual and reproductive health outcomes in adolescent girls and young women in low- and middle-income countries? (2) What specific outcomes are associated with early menarche in these countries? (3) Are the associations found in low- and middle-income countries the same as those found in high-income countries? (4) What are some underlying factors, mediators, and moderators that influence these associations?

## Methods

We report the findings of our systematic review following the Preferred Reporting Items for Systematic Reviews and Meta-Analyses (PRISMA) guidelines [36]. Prior to beginning our review, we developed a protocol to guide the conduct of our systematic review. The study protocol is registered in PROSPERO (CRD42015023349) [37].

### Search strategy

We searched PubMed, PsycINFO, Embase, CINAHL, POPLINE, ProQuest Social Science, Social Sciences Full Text, and Social Sciences Citation Index for peer-reviewed studies that assessed the relationship between menarche and various negative sexual and reproductive health outcomes in adolescence and young adulthood including early sexual debut, experiences of sexual advances from older men, early pregnancy and childbirth, sexual risk taking, unsafe sexual behaviors, unwanted pregnancy, sexual violence, sexually transmitted infections, and HIV. We also searched for studies that assessed the association between age at menarche and early marriage, given that the latter is often a gateway to early sexual behavior, pregnancy and childbearing. We compiled a list of potential search terms identified through preliminary searches, pilot tested them and modified them to improve the thoroughness of our final search. To ensure that all relevant studies were captured, we conducted our final searches using both free text keywords and controlled vocabulary specific to each database. (Only free text keywords were used to search Social Sciences Citation Index because the database does not use controlled vocabulary). We limited our searches to human studies published in English between January 1, 1980 and April 9, 2017. The search strategy used in PubMed is presented in Table 1. In addition to the database searches, we also reviewed the bibliographies of relevant studies for additional citations to evaluate for inclusion in our review.

**Table 1:** PubMed specifications of search strategy for systematic review on early menarche and sexual and reproductive health in low- and middle-income countries.

	Focus	Operator	Search Terms
1	Early menarche	Keywords	((Menarche[Mesh] OR Menstruation[Mesh] OR Menarche*[tw] OR Menstrua*[tw] OR menses[tw]) AND (“Age factors”[Mesh] OR “age of onset”[tw])) OR “Early Menarche”[tw]
2	Sexual and reproductive health	Keywords	"Reproductive Health"[Mesh] OR ((sexual[tw] OR reproducti*[tw])) AND (health[tw]) OR sexual reproducti*[tw]
3	Pregnancy	Keywords	"Pregnancy in Adolescence"[Mesh] OR "Pregnancy, Unplanned"[Mesh] OR (pregnan*[tw]) AND (early[tw] OR adolesc* [tw] OR teen* [tw] OR unplanned[tw] OR unwanted[tw])
4	Sexual behavior	Keywords	((sexual[tw]) AND (behavior*[tw] OR behaviour*[tw] OR risk*[tw] OR partner*[tw])) OR sexuality[Mesh] OR sexualit*[tw] OR "Sex Factors"[Mesh] OR coitus[Mesh] OR coitarche[tw] OR coitus[tw] OR “sexual debut”[tw] OR "Sexual Behavior"[Mesh:NoExp] OR "Risk-Taking"[Mesh:NoExp] OR "Risk-Taking"[tw] OR "Sexual Partners"[Mesh] OR "Condoms"[Mesh] OR Condom*[tw] OR “unsafe sex”[tw] OR “unprotected sex”[tw]
5	Sexually transmitted Infections	Keywords	"Sexually Transmitted Diseases"[Mesh] OR Sexually Transmit*[tw] OR STI[tw] OR STD*[tw] OR "HIV"[Mesh] OR "HIV Infections"[Mesh] OR HIV[tw]
6	Sexual violence	Keywords	"Sex Offenses"[Mesh] OR ((spous*[tw] OR sexual[tw] OR intimate[tw] OR dating[tw] OR gender[tw])) AND (violence[tw] OR coercion[tw] OR victim* [tw])
7	Marriage	Keywords	"Marriage"[Mesh] OR marri*[tw]
8		Boolean operator	#2 OR #3 OR #4 OR #5 OR #6 OR #7
9		Boolean operator	#1 AND #8
10		Limits	#1 AND #8 Filters: Publication date from 1980/01/01 to 2017/12/31; Humans; English

### Eligibility criteria

**Fig 1:**
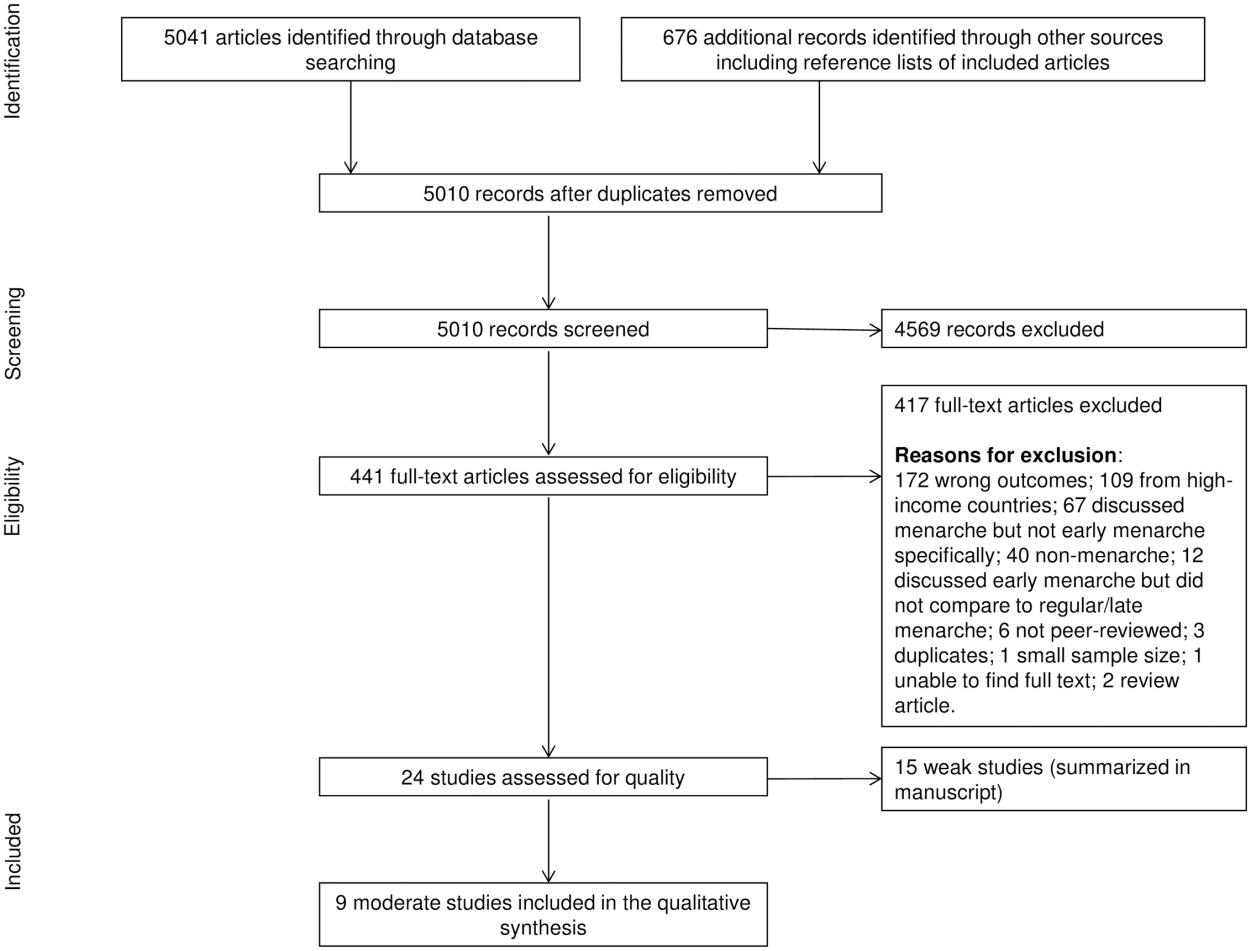
PRISMA flowchart of study selection for systematic review on early menarche and sexual and reproductive health in low- and middle-income countries.

Studies were selected for inclusion in the systematic review through a multi-step process (Fig 1). First, two researchers independently screened the title and abstract of each identified citation for relevance. Next, they each individually reviewed the full texts of each relevant article to ensure that the articles met all eligibility criteria. In each step, the researchers resolved all disagreements through discussion until a consensus was reached. We included studies if they: were conducted in a low- or middle-income country, assessed the association between early menarche and at least one of the sexual and reproductive health outcomes of interest, were peer-reviewed, and published in English since 1980. We excluded case reports and other studies with small sample sizes (n<50 for quantitative studies and n<15 for qualitative studies), due to the fact that such studies might not be representative of the larger population. We also excluded studies that assessed the determinants of early menarche, but not factors that occurred after menarche. Additionally, we excluded studies that assessed the association between early menarche and biological factors pertaining to sexual and reproductive health (other than STIs), such as genetic factors and various forms of cancer.

### Quality and risk of bias assessment

Two researchers independently assessed the quality of each study that met all eligibility criteria using a modified version of the Joanna Briggs Institute’s critical appraisal tools for observational quantitative studies [38]. The Joanna Briggs Institute promotes the translation of evidence into practice through evidence synthesis, and has developed detailed methodology and tools to facilitate systematic reviews including critical appraisal tools for different study designs [38]. Based on the critical appraisal tools, we assessed the studies on nine factors: (1) the appropriateness of the study design given the research question(s), (2) whether the study sample was representative of the stated target population, (3) whether the eligibility criteria were clearly defined, (4) whether participants were followed for a sufficient amount of time (where applicable), (5) whether all people who entered the study were accounted for at its conclusion and in the analysis, (6) the reliability and validity of the outcome measure(s), (7) whether possible confounders were addressed in the analysis and article, (8) whether the comparison groups were clearly defined, and (9) the appropriateness of the statistical analyses used. In addition, we also evaluated the studies on: the appropriateness of data sources used for the study, reliability and validity of key independent variables, the credibility of the results given the methods used, consistency of conclusions with study results, whether or not study limitations were addressed and accounted for when interpreting the results, and whether or not ethical considerations were addressed. Each factor that was assessed was rated as *strong*, *moderate*, *weak*, *unclear*, or *not applicable*. Based on the totality of assessments, we classified articles with no weak or unclear ratings as strong. Articles with up to five weak or unclear ratings were classified as moderate, and articles with six or more weak or unclear ratings were classified as weak.

### Data extraction and analysis

Using a standardized form, two researchers individually extracted data from each included study. The extracted data included: country where the study was conducted, sample size, age range of study participants, study design, definition of early menarche used in the study, groups compared in the study, relevant outcomes examined in the study, and the main findings pertaining to our study questions.

We conducted a thematic analysis of the findings from all included studies and summarized the results by outcomes. We were unable to conduct a meta-analysis due to the heterogeneity of the studies in terms of populations, outcomes assessed, operationalization of early menarche and outcomes, analytical methods used and the small number of high quality eligible studies.

## Results

### Search results and study characteristics

Our search of the literature yielded 5010 unique citations (Fig 1). Twenty-four studies met all inclusion criteria and were assessed for quality and risk of bias. Nine of the twenty-four studies were of moderate quality (Table 2) while the remaining fifteen studies had several methodological weaknesses (Table 3). The quality assessment and main methodological weaknesses of all eligible studies are presented in the last column of Tables 2 and 3. Due to the small number of articles of moderate quality, we also include a brief discussion of the weak studies below.

**Table 2:** Characteristics of the moderate quality studies included in the systematic review on early menarche and sexual and reproductive health in low- and middle-income countries.

Study	Country	Study Design	Sample Characteristics	Menarche Comparison Groups	Relevant Outcomes	Main Findings	Quality of Study
Ajah (2015)[39]	Nigeria	Cross-sectional	482 adolescent secondary school girls from Abakaliki, Nigeria (urban); Ages 13–19	Menarche at or before 13 years of age versus menarche at 14 or older	Sexual activity	Girls who reached menarche at ≤ 13 years were more likely to report being sexually active than those with menarche at age ≥ 14 years (p = 0.0001)	Moderate study. Unclear what was done to minimize non-response. Reliability and validity of measures used to assess the various study variables, and potential confounders were not addressed.
Aryal (2007)[40]	Nepal	Cross-sectional	1566 women (1019 married; 547 unmarried) from Palpa and Rupandehi districts in rural Nepal	Menarche before 13 years of age versus menarche at ages 13–14, 15–16, and 17 or older	Age at first marriage	The median age at marriage increased with increasing age at menarche. Median age at marriage was approximately 16, 17, 17 and 18 years for females whose age at menarche was less than 13, 13–14, 15–16 and 17 years and above respectively. Menarche was significantly associated with girls’ age at first marriage (χ^2^ = 16.55; df = 3; p = 0.0009) in univariate hazard models. Those whose menarche occurred at the ages of 13–14, 15–16 and 17 years and above had, respectively, 15%, 23% and 44% less risk of getting married early compared to those whose menarche occurred before the age of 13 years.	Moderate study. Did not adequately specify inclusion criteria, or address potential confounders and study limitations.
Buga et al. (1996)[41]	South Africa	Cross-sectional	1072 adolescent girls in standards 5–7 in Transkei, Eastern Cape, South Africa; mean age 15.29 ±1.89	Age at menarche was assessed as a continuous variable	Age at first sexual intercourse, lifetime number of sexual partners, age at first pregnancy	Age of menarche was positively correlated with the age at first coitus (r = 0.26; 95% CI: 0.15–0.36). There was no correlation between the age of menarche and the lifetime number of sexual partners. Age of menarche was positively correlated with the age at first pregnancy (r = 0.41; 95% CI: 0.20–0.59)	Moderate study. Reliability and validity of measures used to assess the various study variables, and what confounders controlled for unclear.
Field (2008)[42]	Bangladesh	Cross-sectional	2101 ever married women from Matlab, Bangladesh (rural); Ages 25–44	Age at menarche was assessed as a continuous variable	Age at first marriage	Mean age at first marriage increased with age at menarche. Mean age at marriage was 14.71, 15.72, and 16.73 years for women with menarche at ages 11–13, 14, and 15–17 years respectively. For each additional year that menarche is delayed, the age of marriage increases by 0.74 year (*F*-statistic = 146; standard error = 0.06; p<0.01) More than 70% of first marriages occur within 2 years of menarche. Less than 18% of women were married before menarche; 68% of these marriages preceded menarche by 1 year.	Moderate study. Unclear what was done to minimize non-response.
Glynn et al. (2010)[43]	Malawi	Cross-sectional	6825 women from Karonga District, Malawi (rural); Ages 15–59	Menarche before 14 years of age versus menarche at ages 14–15, and 16 or older	Age at first sexual intercourse, age at first marriage	Age at menarche was associated with age at sexual debut, with 55% of those with menarche at <14 years having had early sex before age 16, compared with 27% of those with menarche at age 14–15, and 4% of those with menarche aged ≥16 years. The median interval between menarche and sexual debut was 3.5 years for those with menarche age <14, 2.7 years for those with menarche at 14–15, and 2.5 years for those with menarche at 16 or older. Earlier age at menarche was associated with younger age at first marriage, with those with menarche <14 years having a median age at marriage of 16.9 versus 18.5 and 20.3 among those with menarche at 14–15 years and ≥16 years respectively.	Moderate study. Reliability and validity of measures used to assess the various study variables not adequately addressed. Unclear what was done to minimize non-response.
Glynn et al. (2014)[44]	Malawi	Cross-sectional	3965 women from Karonga District, Malawi (rural); Ages 15–30	Menarche before 14 years of age versus menarche at ages 14, 15, and 16 or older	Age at first sexual intercourse, age at first marriage, STIs (HIV & HSV-2)	Earlier age at menarche was associated with the earlier age at sexual initiation. Age at first marriage increased with increasing age at menarche. Those with menarche at age 14 or older were 11–31% less likely to test positive for herpes simplex type 2 virus (HSV-2) compared to those with menarche before age 14 after adjusting for age. Age at menarche was not associated with HIV infection.	Moderate study. Inclusion criteria not clearly stated. Reliability and validity of measures used to assess the various study variables unclear.
Patel (2012)[45]	Zimbabwe	Cross-sectional	200 HIV-positive women in urban Zimbabwe; Age 18+ (range: 22–69)	Age at menarche was assessed as a continuous variable	HIV serostatus disclosure	The mean age at menarche for women who disclosed their HIV sersostatus to their current sexual partner was 14.6 years compared to 13.8 years for women who did not disclose (p = 0.02) In univariate analysis, age at menarche was a statistically significant predictor of HIV disclosure (OR = 0.74; 95% CI: 0.56–0.97). Age at menarche was a marginally significant predictor of HIV disclosure in multivariate analysis (OR = 0.71; 95% CI: 0.51–1.00)	Moderate study. Did not discuss potential confounders or efforts to minimize non-response.
Sekhri (2014)[46]	India	Cross-sectional	5787 ever-married mothers; Ages 15–49.	Menarche was assessed as a continuous variable	Age at marriage	Age at menarche was a significant predictor of age at marriage (β = 0.32; p<0.01). The association between age at menarche and age at marriage remained statistically significant after controlling for birth year, height and the asset index of the husband’s family (β: 0.2–0.3; p<0.01).	Moderate study. Study limitations were not addressed.
Wyatt et al. (1999)[47]	Jamaica	Cross-sectional	897 women ages 15–50 drawn from a nationally representative probability sample	Age at menarche was assessed as a continuous variable	Age at first sexual intercourse	Women who reported later ages of menarche were 28% less likely than those with earlier menarcheal ages to engage in intercourse before age 16.	Moderate study. Study limitations not adequately addressed and accounted for when interpreting findings

**Table 3:** Characteristics of the weak quality studies included in the systematic review on early menarche and sexual and reproductive health in low- and middle-income countries.

Study	Country	Study Design	Sample Characteristics	Menarche Comparison Groups	Relevant Outcomes	Main Findings	Quality of Study
Islam, et al. (1996) [48]	Bangladesh	Cross-sectional	11906 (8,467 rural and 3,439 urban) ever-married Bangladeshi women under the age of 50	Menarche was assessed as a continuous variable	Age at first marriage; timing of marriage in relation to menarche	Among women who were married before age 20 (96% of the study sample), 18.1% were married before menarche, 18% were married at the same time as menarche, and 63.9% were married after menarche. Marriage occurs shortly after or around the time of menarche among women who married before age 20, with a mean age at menarche of 13.4, mean age at marriage of 14.5, and mean age at marriage consummation of 14.8	Weak study. No information provided on the measures used to assess the various study variables or the specific statistical analyses used. Study limitations, possible confounders and ethical considerations not addressed.
Kolachi, et al. (2013) [49]	Pakistan	Cross-sectional	129 women residing in Tharparkar Desert, ages 11–50 years old.	Unclear (mean age at menarche 13; range 11–17)	Age at marriage, age at first pregnancy	Early menarche and early marriage have increased the span of pregnancy	Weak study. Minimal details provided on study methodology. Representativeness of the study population and eligibility criteria unclear. No information provided on the measures used to assess the various study variables. Possible confounders, study limitations and efforts made to increase response rate not discussed. Conclusions not consistent with results provide.
Kumar (2008)[50]	India	Not specified	1122 currently married women with a live birth in the last 5 years in (urban, rural and coastal) Thiruvananthapuram, Kerala; Ages 16–45	Menarche before age 12 years, menarche at 12–15 years, or menarche older than 15 years	Waiting time to first birth after menarche	Women with early menarche (<12 years) had a longer waiting time between menarche and first birth compared to women with later menarche (p<0.001). The odds of waiting more than eight years between menarche and motherhood was 21.08 (95% CI: 9.33–47.61; p<0.001) for those with early menarche (<12 years), and 3.71 (95% CI: 1.99–6.91; p<0.001) for those with normal puberty (12–15 years), compared to women with delayed menarche (≥15 years).	Weak study. Reliability and validity of measures used to assess the various study variables, study limitations, and ethical considerations were not discussed. Study design, inclusion criteria, and what was done to minimize non-response are unclear.
Lema (1990) [51]	Kenya	Cross-sectional	1751 secondary school girls in Nairobi, ages 12–19	Menarche was assessed as a continuous variable (range 10–17)	Age at first coitus in relation to age at menarche	62.3% of sexually active girls started having sexual intercourse within 1 to 2 years of menarche. About 16% of the sexually active girls reported an age at first sex that was lower than their age at menarche.	Weak study. No information provided on the measures used to assess the various study variables or the statistical analyses used. Representativeness of study sample is unclear. Ethical considerations, possible confounders, and study limitations not addressed.
Raj (2015)[52]	India	Cross-sectional	1062 married women in rural Maharashtra, India; Ages 18–30	Menarche at age 8–12, 12.1–13, 13.1–14, or 14.1–18 years	Age at marriage	After adjusting for demographics and gendered risks, women with menarche at ages 8–12 were significantly more likely than those with menarche ≥14.1 to get married at ≤ 15 years of age (aOR = 4.36; 95% CI: 1.68–11.32), but not at 16–17 years (aOR = 1.51; 95% CI: 0.92–2.48) Women with menarche at ages 12.1–13, were significantly more likely than those with menarche ≥14.1 to get married at ≤ 15 years of age (aOR = 4.00; 95% CI: 1.63–9.82), and at ages 16–17 years (aOR = 1.58; 95% CI: 1.03–2.42) after adjusting for demographics and gendered risks. Post-hoc analysis found a significant interaction between age at menarche and education (p<0.001), with those with earlier menarche and less education being more likely to get married at an early age.	Weak study. Several factors are unclear, including inclusion criteria, representativeness of the study sample, efforts to minimize non-response, and what confounders were controlled for in the analysis. The reliability and validity of the measures used were not discussed.
Reddy (2010)[53]	India	Not specified	200 women from the Setti Balija caste in Andra Pradesh India	Age at menarche was assessed as a continuous variable	Age at marriage	Age at menarche is strongly correlated with age at marriage, with the mean age at marriage increasing from 13.69 for those with menarche at age ≤12 to 17.51 for those with menarche at age 16+. Most girls were married within two years of menarche.	Weak study. Several factors are unclear, including the study design, inclusion criteria, the age group of the participants, representativeness of the study sample, efforts to minimize non-response, whether and how potential confounders were accounted for. Study limitations and the validity and reliability of the measures used to assess the various study variables were not discussed.
Riley (1994) [54]	Bangladesh	Longitudinal and Cross-sectional	Sample size not specified–nearly 1500 girls ages 10–20 in rural Bangladesh completed the baseline, about 70% of those completed the follow up cross-sectional survey	Menarche was assessed as a continuous variable	Age at marriage, age at first birth	Lower age at menarche was associated with younger age at marriage, with a median age at marriage of 14.7 years for those with menarche at age 12 versus 20.1 years for those with menarche at age 17. More than 10% of girls were married before menarche. The menarche to marriage interval was not dependent on age at menarche.	Weak study. Several factors are unclear, including sample size, efforts to increase response rate, statistical analysis used, and potential confounders controlled for in analyses. Study sample not representative of rural Bangladesh. Those lost to follow-up were not accounted for. Ethical considerations not addressed
Schor (1993) [55]	Brazil	Not specified	2588 women ages 12–55 in urban Brazil (Santo André), with a history of abortion	Menarche at ages 9–12, 12–14, and 15 or above (mean age at menarche 13.4; range 9–19 years)	Age at the first sexual relation, age at the first pregnancy	In women with a first pregnancy, age at menarche was significantly correlated to both age at first sexual relation (r = 0.22) and age at first pregnancy (r = 0.15). Among those pregnant for the first time under the age of 20, age at menarche was significantly correlated with both age at first sexual relation (r = 0.39) and age at first pregnancy (r = 0.28). Among those pregnant for the first time over the age of 20, age at menarche was significantly correlated with at first sexual relation (r = 0.11).	Weak study. Study design, specific statistical analyses used, reliability and validity of measures used to assess the various study variables, representativeness of study sample are all unclear. Ethical considerations, efforts to increase study response rates, possible confounders and study limitations not addressed.
Shayan (2014)[56]	Iran	Cross-sectional	654 women from rural areas of Shiraz in Southern Iran; Ages 15–49.	Menarche ≤13 versus >13. Menarche was also assessed as a continuous variable	Interval from marriage to first birth	The interval to first birth did not significantly differ between women with menarche ≤13 compared to those with menarche >13 (p = 0.37). In survival analysis, age at menarche was not a significant predictor of time to first birth (HR = 1.01; p = 0.67)	Weak study. The representativeness of the study sample, efforts to minimize non-response, validity and reliability of measures used, potential confounders and study limitations were not discussed.
Sureender, et al. (1998) [57]	India	Cross-sectional	3948 ever married women, ages 13–49	Menarche below age 13, 13–14, over 14	Age at marriage, age at first birth	In multivariate analyses, age at menarche had a significant influence on age at marriage. Those with older age at menarche were married at an older age as compared to women with lower age at menarche.	Weak study. Eligibility criteria unclear. Ethical considerations, possible confounders, study limitations, efforts taken to increase response rate, and reliability and validity of measures used to assess the various study variables not addressed.
Tan Boon, et al. (1983) [58]	Malaysia	Cross-sectional	1252 ever married women; ages 25–50 years	Menarche was assessed as a continuous variable	Age at first marriage and age at first birth	Each year of increase in age at menarche was associated with a three-month increase in age at marriage, after controlling for ethnicity. After controlling for ethnicity, birthdate, socioeconomic level and living abroad during childhood, each year of increase in age at menarche was associated with a five-month increase in age at marriage Age at menarche was similarly associated with age of first birth after controlling for ethnicity and birthdate.	Weak study. Insufficient information provided on specific statistical analyses used, and reliability and validity of measures used to assess the various study variables. The sample was not representative of the population of interest. Does not address limitations, ethical considerations or efforts to increase response rate.
ter Meulen, et al. (1992) [59]	Tanzania	Cross-sectional	53 cancer patients (age 21–91 years); 359 non-cancer patients (age 15–70 years)	Menarche at age below age 13, 13–16, above 16 years	STIs—HPV and HIV	27.3% of the women with menarche below 13 years of age were HPV-16/18-positive, compared to 17.6% among those with menarche between ages 13 and 16 and 6.5% in those with menarche above age 16. Compared to those with menarche above age 16, the age adjusted odds ratio of HPV 16/18 infection was 6.00 (p = 0.02) for those with menarche below age 13 and 3.25 (p = 0.05) for those with menarche between 13 and 16 years. After adjusting for other covariates, the odds ratio of HPV 16/18 infection for those with menarche below age 13 was 6.2 (p = 0.03), while that of those between ages 13 and 16 was no longer statistically significant. Age at menarche was not significantly associated with HIV and other types of HPV.	Weak study. Eligibility criteria, sample size, representativeness of study sample, and confounders controlled for in analyses are all unclear. Does not address limitations, ethical considerations or efforts to increase response rate.
Udry & Cliquet (1982) [60]	Pakistan (subsample)	Unspecified	200 women; age unspecified	Menarche at 226412, 13, 14, 15, and ≥16 years for one analysis; and menarche at ≤11, 12, 13, 14, and ≥15 years for the other analysis	Age at marriage, age at first birth	By age 17, 85% of those with menarche at ≤12 years were married compared to 10% among those with menarche at ≥16 years. The mean age at first marriage was 15.24 years for those with menarche at age 12 versus 19.65 years among those with menarche at age ≥15. The mean age at first birth ranged from 16.82 years among those with menarche at age 12 to 21.19 years among those with menarche at age ≥15.	Weak study. Eligibility criteria, statistical analyses used, and confounders controlled for in analyses are all unclear. No information provided on the measures used to assess the various study variables. The sample was not representative of any population. Does not address ethical considerations or efforts taken to increase response rate.
Udry & Cliquet (1982) [60]	Malaysia (subsample)	Cross-sectional?	1018 women under age 50	Menarche at ≤12, 13, 14, 15, and ≥16 years for one analysis; and menarche at ≤11, 12, 13, 14, and ≥15 years for the other analysis	Age at marriage, age at first birth	Among ethnic Malay women, 70% of those with menarche at ≤12 were married by age 16 compared to 20% among those with menarche at ≥16. The mean age at first marriage ranged from 16.19 years for those with menarche ≤11 to 18.10 years among those with menarche at ≥15 years, among Malay women. Among Malay women, 65% of those with menarche at ≤12 were married by age 18 compared to 25% among those with menarche at ≥16 years. The mean age at first birth ranged from 18.68 years for those with menarche ≤11 to 20.22 years among those with menarche at ≥15 years, among Malay women Among Malaysian Chinese women, earlier age at menarche was only associated with earlier age at marriage or first birth after controlling for education.	Weak study. Eligibility criteria, statistical analyses used are unclear. No information provided on the measures used to assess the various study variables. Does not address ethical considerations or efforts taken to increase response rate.
Varea (1993) [61]	Morocco	Not specified	842 married women living in Amizmiz, Marrakech	≤13, 14–15, and ≥16 years,	Age at first marriage, waiting time to first live birth	Age at menarche was significantly correlated with age at first marriage (r = 0.21; p≤0.001) among women under the age of 45, but not among those aged 45 or older. Age at menarche was significantly correlated with age at first live birth both among women under the age of 45(r = 0.21; p≤0.001) and among those aged 45 or older (r = 0.16; p≤0.05). Age at menarche was not significantly correlated with waiting time to first birth in either group of women.	Weak study. Study design and eligibility criteria unclear. Ethical considerations, possible confounders, study limitations, efforts taken to increase response rate, and reliability and validity of measures used to assess the various study variables not addressed.
Varea, et al. (1993) [62]	Morocco	Not specified	496 traditional Women in Amizmiz, Marrakech; aged 25–54	Early menarche: 13 years and under; medium menarche: 14–15 years; late menarche: 16 years and older	Age at first marriage, menstrual age (the difference between age at marriage and age at menarche), waiting time to first live birth	Age at menarche was significantly correlated with age at marriage (0.29; p<0.01) and menstrual age (r = -0.27; p<0.01). Age at menarche was not significantly correlated with waiting time to first birth. Mean age at marriage increased significantly with age at menarche (17.8 years among those with menarche before age 13, 18.0 years among those with menarche at ages 13–15, and 19.7 years among those with menarche at 16 years or older). Menstrual age is significantly shorter for those with late menarche (3.2 years) compared to those with early (4.9 years) or medium menarche (4.5 years).	Weak study. Eligibility criteria, study design, and reliability and validity of measures used to assess the various study variables unclear. Inconsistencies in definition of menarche categories. Limitations, possible confounders, ethical considerations and efforts to increase response rate not discussed.

### Moderate quality studies

Two of the included studies were conducted in Malawi [43, 44]; the others were conducted in South Africa [41], Nepal [40], Jamaica [47], Nigeria [39], Zimbabwe [45], India [46] and Bangladesh [42] (Table 3). All studies were quantitative in nature and utilized cross-sectional designs. Five of the studies assessed age at menarche as a continuous variable [41, 42, 45–47], while the remaining four assessed it as a categorical variable [39, 40, 43, 44]. The mean age at menarche differed by study population and location. Among the sample of Nigerian adolescents, the mean age at menarche was 13.31 [39]; it was slightly higher among the Jamaican women at 13.4 years [47] and the South African sample at 13.9 years [41]. The median age of menarche in one of the studies from Malawi was 15.1 years [43]. No overall measure of central tendency was provided for the other study from Malawi [44], or the studies from India [46], Bangladesh [42] and Zimbabwe [45]. Approximately 25% of participants in the second study from Malawi had menarche at <14, 14, 15, and ≥16 respectively [44]. Among the sample from Nepal, 51% went through menarche at age 13 or 14 [40]. The studies assessed a range of outcomes including early sexual debut, early marriage, early pregnancy, sexual risk-taking and sexually transmitted infections.

#### Early menarche, sexual initiation and sexual activity

Four of the included studies (one from Jamaica, one from South Africa and two from Malawi) explicitly assessed the relationship between early menarche and age at sexual initiation [41, 43, 44, 47], and found that early menarche was associated with an earlier age of sexual initiation. For example, among a sample of Jamaican women, Wyatt et al. found that those with earlier age at menarche were 28% more likely to engage in sexual intercourse before the age of 16 (*p* < 0.0001) [47]. Similarly, among a sample of women in rural Malawi, 55% of those who experienced menarche before age 14 had sex before the age of 16, compared with 27% of those with menarche at age 14–15, and only 4% of those with menarche at age 16 or older [43]. Few girls engaged in sexual intercourse before menarche; for example, less than 3% of study participants in one Malawi study reported sexual intercourse before menarche [43].

Although those with earlier menarche in one study from Malawi were more likely to engage in sexual intercourse at an earlier age, they had a longer duration of time between menarche and sexual initiation compared to those with later menarche. For example, the median interval between menarche and sexual initiation was 3.5 years for those who reached menarche before age 14, compared to 2.7 years for those with menarche at age 14–15, and 2.5 years for those with menarche at age 16 or older [43].

Also, among a sample of adolescent girls aged 13–19 from Nigeria, those with menarche at or before age 13 were significantly more likely to be sexually active than those with menarche at age 14 or older (p = 0.0001); although actual age at sexual initiation was not reported in the study [39].

#### Early menarche and marriage

Five studies (two from Malawi and one each from Nepal, India, and Bangladesh) examined the relationship between age at menarche and age at first marriage [40, 42–44, 46]. Overall, those with earlier age at menarche were more likely to get married at an early age. In the study from Nepal, compared to those who reached menarche before age 13, those with menarche at ages 13–14, 15–16, and 17 years and older were 15%, 23% and 44% less likely to get married early respectively, in multivariate analyses. (However, the association was only statistically significant for those who reached menarche at age 17 or older) [40]. Similarly, in one of the studies from Malawi, the median age at marriage was 16.9 years for those who reached menarche before age 14, compared to 18.5 years among those who reached menarche at 14–15 and 20.3 years among those who reached menarche at 16 years or older [43]. In the study from India, the authors found that for each year that menarche was delayed, the age at marriage increased by 0.74 years (p<0.01) [42].

#### Early menarche and pregnancy

Only one of the included studies assessed the relationship between early menarche and early pregnancy [41]. The study among South African adolescents found a statistically significant positive correlation between the age at menarche and the age at first pregnancy (r = 0.41; 95% CI 0.20–0.59). Thirty-one percent of study participants with data on pregnancy reported having ever been pregnant, with a mean age at first pregnancy of 16.5 years.

#### Early menarche and sexually transmitted infections (STIs)

Two studies examined the relationship between age at menarche and STIs. In one of the studies from Malawi, early menarche was associated with an increased likelihood of infection with Herpes simplex type-2 virus (HSV-2). Those who reached menarche at age 14 or older were 11–31% less likely to test positive for HSV-2 than those who reached menarche before age 14, after adjusting for age. In contrast, age at menarche was not significantly associated with HIV infection [44]. In a study of HIV-positive women from Zimbabwe, those with a later age at menarche were significantly more likely than those with earlier age at menarche to disclose their HIV serostatus to their current sexual partners (p = 0.021) [45]. In the study, later age at menarche was significantly correlated with later age at sexual initiation (ρ = 0.33, p<0.0001). However, since age at sexual initiation was not associated with HIV disclosure to partners, the authors concluded that it was unlikely that the association between age at menarche and HIV disclosure was driven by later age at sexual initiation and greater emotional maturity [45].

#### Early menarche and sexual risk behavior

None of the included studies assessed the association between early menarche and sexual risk behavior in detail. However, the authors of the South African study reported no correlation between the age at menarche and the lifetime number of sexual partners in their sample of adolescent girls [41]. This might have been due to the limited variability in the number of sexual partners the participants had been involved with given their young age; 92.6% of the girls reported having had 1–2 partners, 4.7% reported 3–4 partners, and only 2.7% reported 5 or more partners.

### Weak quality studies

From the articles that were found to be methodologically weak, four studies were conducted in India [50, 52, 53, 57], and two studies each were from Bangladesh [48, 54], Pakistan [49, 60], Malaysia [58, 60], and Morocco [61, 62]. (One of the articles [60] reported results separately for a Pakistan and a Malaysia subsample; these sub-samples are included separately in the study count thus yielding a total of 16 weak studies). The remaining studies were conducted in Brazil [55], Kenya [51], Tanzania [59], and Iran [56]. Most of the studies were cross-sectional [48, 49, 51, 52, 56–59]; one reported using both longitudinal and cross-sectional data [54]. The study design was not specified for six of the studies [50, 53, 55, 60–62]. Most studies assessed age at menarche as a categorical variable [55, 57, 59–62]; four assessed it as a continuous variable [48, 51, 54, 58]. It was unclear how menarche was assessed in one article [49]. The studies looked at several sexual and reproductive health outcomes including early marriage, early childbirth, early pregnancy, early sexual initiation, waiting time (from marriage) to first birth, STIs, and menstrual age (defined as the difference between age at menarche and age at marriage).

Similar to what was found in the moderate studies, those with earlier age at menarche were more likely to get married at an early age [54, 57, 58, 60–62]. For example, Raj and colleagues found that the odds of getting married at age 15 or younger were significantly greater for Indian women who reached menarche at ages 8–12 years (OR = 4.36; 95% CI: 1.68–11.32) and 12.1–13 years (OR = 4; 95%CI: 1.63–9.82) compared to those with menarche at age 14.1 years and older after controlling for demographic and gendered risks [52]. However, two of the studies also reported that a small proportion (18% and 10%) of girls respectively were married before menarche [48, 54]. Earlier age at menarche was also associated with a longer interval between menarche and marriage compared to those with later age at menarche [62]. However, age at menarche was not significantly associated with waiting time from marriage to first birth [56, 61, 62].

Earlier age at menarche was associated with earlier age at pregnancy, and at first live birth [55, 58, 60, 61]. However, in India, earlier age at menarche was also associated with increased odds of having more than an eight-year duration from menarche to first motherhood; the odds for those with menarche before age 12 years was 21.08 (95% CI: 9.33–47.61) and that of those with menarche at ages 12–15 years was 3.71 (95% CI: 1.99–6.91) compared to women with menarche over the age of 15 years [50].

As was the case with the moderate studies, early menarche was associated with an earlier age of sexual initiation [55]. However, sexual initiation prior to menarche was more common in the weak studies, with Lema and colleagues reporting that 16% of girls in the study from Kenya engaged in sexual intercourse before menarche [51]. In the study from Tanzania, early menarche was associated with infection with HPV types 16/18, but not with HIV or other types of HPV [59].

## Discussion

Despite the vast body of research on adolescent sexual and reproductive health in low- and middle-income countries, our systematic review shows that very little is known about the associations between early menarche and sexual and reproductive health outcomes in such settings. The review highlighted that early menarche is associated with early marriage, early sexual initiation, early pregnancy and childbirth, and some STIs in lower-income regions of the world; similar to what has been observed in high-income countries. We found limited research on the association between early menarche and sexual risk behavior. However, it is unclear whether this is due to insufficient research on the topic to date or that the association does not exist. Despite similarities in the relationship between early menarche and sexual and reproductive health in low-, middle- and high-income countries, the underlying factors driving these associations might not be the same due to differences in sociocultural contexts and structural factors across and within such countries. Given the small number of eligible studies and the study designs utilized, we were limited in our ability to determine the underlying factors, mediators and moderators of the associations identified.

Of significance, our review highlighted the association between early menarche and early marriage (the outcome assessed in the majority of eligible studies). This finding could be particularly relevant to ongoing global efforts to curb child marriage. In the study from Nepal, Aryal noted that girls from certain ethnic groups may be married off before menarche for cultural or religious reasons [40]. Similarly, two of the weaker studies from Bangladesh found that 10–18% of girls were married before menarche [48, 54]. Continued declines in age of menarche in these regions may lead to girls being married off at even younger ages. In other regions of the world, menarche signals a readiness for marriage, with girls being married off shortly after they reach menarche [43, 44]. The association between early menarche and early marriage, as well as the related factors, may differ across ethnic groups within the same country [58, 60]. This highlights the need for a greater understanding both within and between countries of the cultural and regional variations in the effect of age at menarche on age at marriage. More research may provide important insights to bolster current efforts to end child marriage and improve the sexual and reproductive health outcomes of adolescent girls and young women.

Although not assessed in the studies included in our review, research suggests that age at first marriage may mediate the association between early menarche and other sexual and reproductive health outcomes. For example, early marriage may increase girls’ vulnerability to the negative health effects of early pregnancy and childbirth, since girls are often expected to bear children soon after marriage [12, 63]. In addition, girls who get married early are often unable to effectively negotiate safer sex, increasing their vulnerability to STIs such as HIV [64]. This is evidenced by research from Kenya and Zambia, which shows considerably higher rates of HIV infection among ever-married girls aged 15–19 years compared to their unmarried but sexually active counterparts [12]. Furthermore, girls who are married in adolescence are more likely than those who marry at a later age to experience sexual violence [1, 63, 65].

Despite decreasing rates of child marriage worldwide, 280 million girls today are still at risk of becoming child brides [64]. In particular, girls living in poor and rural areas of South Asia and sub-Saharan Africa experience high rates of child marriage [63, 64]. In Niger–which has the highest prevalence of child marriage in the world–75% of women aged 20–24 were married before the age of 18 in 2006, reflecting a minimal decline from 77% in 1998 [63]. Thus, our findings suggest that child marriage prevention efforts may benefit from targeting programs and policies to early-maturing girls, focusing on areas where girls reach menarche at younger ages and in regions where menarche signals readiness for marriage. Due to varying cultural beliefs regarding early marriage both across and within countries, such efforts should be tailored to the specific context.

Several of the included studies reported an association between early menarche and early sexual initiation. This association is well documented in the research literature from high-income countries [14, 18–22]. Early sexual initiation has important implications for the overall health of young women, and may also be a key driver of associations observed between early menarche and early pregnancy and STIs. Although none of the studies included in our review expressly examined early sexual initiation as a mediator of the association between early menarche and sexual and reproductive health, some studies suggest that this might be the case. For example, in the study from South Africa, Buga et al. found a much stronger correlation between age at first sex and age at pregnancy (r = 0.68) than between age at menarche and age at pregnancy (r = 0.41) [41]. This suggests that early sexual initiation among those with early menarche might be driving the association between early menarche and early pregnancy.

Adolescents who initiate sexual intercourse at a young age are often vulnerable to coerced, risky and unprotected sex [66–68]. Unprotected sexual activity may in turn lead to unwanted pregnancy and childbearing, abortion, or infection with HIV and other STIs [69]. Girls with early menarche who initiate sexual activity at a young age may not be developmentally prepared for sex (both mentally and physically) or knowledgeable about taking precautions to prevent pregnancy and STIs when having sex [66]. High rates of unmet need for contraception in low- and middle-income countries further limit young girls’ abilities to prevent early pregnancies [5]. In addition, the underdeveloped vaginal epithelial lining in younger girls is more vulnerable to tears during sexual intercourse than that of adult women, thus increasing young girls’ risks of contracting STIs including HIV [70–72].

Structural factors may also underlie the association between early menarche and sexual and reproductive health outcomes. Due to gender inequalities, girls are often unable to insist that their sexual partners take the necessary precautions to prevent pregnancy and STIs during sex [65]. They are frequently unable to decide when to have sex for the first time, with younger girls even less likely to have negotiating power within such gendered dynamics [65]. For example, in some parts of sub-Saharan Africa, 45% of girls report that their first sexual intercourse was forced [65]. Girls who reach menarche at an early age may be especially vulnerable, as they may be perceived as sexually mature due to their more adult-like physical appearances [73]. Despite this increased vulnerability, young girls have little access to necessary sexual and reproductive health services, leading to possible long-term morbidity [1]. These factors may explain the associations found between early menarche and early pregnancy and STIs both in our systematic review and in studies from high-income countries [14, 17, 18]. Although early menarche was not found to be associated with HIV infection, its association with other STIs and the decreased likelihood of HIV serostatus disclosure are still of particular concern in countries with generalized HIV epidemics–such as many countries in sub-Saharan Africa–where the age at menarche has also been declining over the past few decades [30, 31, 74, 75]. All these findings highlight the need for better data on age at menarche, and for the inclusion of menarche indicators in nationally-representative surveys such as the Demographic and Health Surveys (DHS), UNICEF’s Multiple Indicator Cluster Survey (MICS) and Performance Monitoring and Accountability 2020 (PMA2020) [13].

Despite these findings, several questions remain unanswered, indicating a need for further research in this area. First, none of the studies included in our review examined the effect of other pubertal changes on sexual and reproductive health. Early menarche is the most commonly used indicator of early sexual maturation since menarche occurs late in the pubertal development sequence and is one of the more salient aspects of puberty for girls [25, 76]. However, other physical and hormonal changes that accompany puberty, including breast development, occur prior to menarche [25] and may play an important role in explaining the association between early menarche and these outcomes [60]. This may especially be the case in societies such as northern Ethiopia, where girls often hide the onset of menarche from others [77]. Girls with more mature looking physiques–with developed breasts and hips–may be more vulnerable to sexual advances from their male peers and older men [73, 78]. In addition, hormonal changes that accompany puberty may lead early-maturing girls to experience sexual desires and engage in sexual behavior at a young age [60, 78, 79]. The pubertal changes that occur before menarche might explain the findings from the studies from Malawi and Kenya, indicating that some girls engage in sexual intercourse before menarche [43, 51].

Second, due to the cross-sectional nature of the included studies, it is impossible to establish the temporal order of the observed associations–that is, whether early menarche precedes the observed outcomes. For biological reasons, some outcomes such as pregnancy and childbirth must be preceded by menarche. However, that is not the case with STIs and early sexual initiation. In one study from Malawi, Glynn et al. noted that only 2.8% of girls engaged in sexual intercourse before menarche, suggesting that for most girls, menarche occurs before first sex [43]. In contrast, the study from Kenya found a larger proportion of girls who had sex before menarche (16%) [51]. No comparisons were made between girls who engaged in sexual intercourse before menarche and those who did not in either study. Yet, such comparisons may provide important insights to support efforts to mitigate vulnerability to negative sexual and reproductive health outcomes in adolescence and young adulthood.

### Limitations

First, in an attempt to establish the associations between early menarche and sexual and reproductive health in low- and middle-income countries using only the most rigorous scientific evidence available, we limited our systematic review to peer-reviewed articles. Thus research reports and other gray literature that may have examined the associations between early menarche and these outcomes, and might provide further insights, were excluded from our review. Second, by limiting our review to articles published in English, we may have missed additional articles. This may account for the fact that the review only identified one (weak) study from South America [55]; results from that region may have been published in Spanish or Portuguese. Third, since our goal was to determine whether early menarche might be an important factor that needs to be considered in efforts to decrease adolescent girls’ vulnerability to negative sexual and reproductive health, we did not assess ways in which early menarche, sexual activity and reproduction might be beneficial to girls nor did we look at positive outcomes associated with early menarche. Finally, by limiting our review to only sexual and reproductive health outcomes, we may have missed relevant studies on psychosocial factors associated with early menarche. This may have hindered our ability to better understand the underlying factors driving the association between early menarche and our outcomes of interest.

Studies from several high-income countries have shown that early menarche is associated with various psychosocial factors including delinquency, substance use, and depression [14, 19, 26, 80–88], all of which have sexual and reproductive health implications. Research from high-income countries has also shown that early menarche may be linked to higher school dropout rates [17], suggesting that early menarche may have indirect economic costs that are not apparent when focused solely on health outcomes. Findings from our review suggest that similar patterns may also be found in low- and middle-income countries. One of the studies from Malawi, found that educational attainment was associated with age at menarche, with only 46% of girls with menarche before age 14 completing primary school, compared to 60% and 70% among girls with menarche at ages 14–15 and 16 and older respectively [43]. Furthermore, the Malawi study indicated that age at sexual initiation and age at first marriage both partially explained the association of menarcheal age with educational attainment. Women in the study cited marriage and pregnancy (but not menstruation) as their main reasons for discontinuing school [43]. Udry and Cliquet’s findings from Malaysia also suggest that education may be an important confounder of the menarche-sexual and reproductive health association in some groups [60]. These findings suggest that the association between early menarche and sexual and reproductive health may be affected by and/or affect various psychosocial and socioeconomic factors, which warrant further exploration in low and middle-income countries.

### Implications for future research

Our systematic review indicates that early menarche may be an important factor influencing the sexual and reproductive health of adolescent girls and young women in low- and middle-income countries. Based on our findings, we recommend longitudinal studies that can capture girls’ transitions from childhood into young adulthood to confirm the existence of the identified associations across different settings. Such research may strengthen our understanding of how various psychosocial, behavioral, and demographic factors change over time and affect the sexual and reproductive health of girls as they reach puberty and transition into adulthood. These studies may also elucidate the influence of context-specific social and cultural norms on girls’ health outcomes, especially in relation to menarche and puberty. Such studies could be particularly helpful for teasing out the temporal order in which menarche and negative sexual and reproductive health outcomes occur in order to identify critical points of intervention. Finally, we recommend that the global public health community take into consideration the declining age at menarche in low- and middle-income countries, particularly when designing and implementing research, programming and policy aimed at ending child marriage and improving adolescent health outcomes. More specifically, early-maturing girls and regions with earlier ages of menarche should be targeted for intervention. By strengthening our understanding on the effect of early menarche on sexual and reproductive health, we may identify new approaches, including the timing of intervention delivery to more effectively address the high burden of negative sexual and reproductive health outcomes still experienced by adolescent girls and young women across low- and middle-income countries.

## Supporting information

S1 Protocol Review protocol for the systematic review on early menarche and sexual and reproductive health in low- and middle-income countries. (DOCX)

S1 Table PRISMA checklist for the systematic review on early menarche and sexual and reproductive health in low- and middle-income countries. (DOC)
