# Supplementary material for: Early menarche: A systematic review of its effect on sexual and reproductive health in low- and middle-income countries
Source: PLoS One. 2017 Jun 7;12(6):e0178884. doi: 10.1371/journal.pone.0178884 (PMC5462398; doi:10.1371/journal.pone.0178884)
Supplement: S1 Protocol — (DOCX) [file pone.0178884.s001.docx]

**Systematic Review Protocol**

**Title:**

Associations between early menarche and various sexual and reproductive health outcomes in low- and middle-income countries

**Review question:**

Is early menarche associated with negative sexual and reproductive health (SRH) outcomes in young women?

What negative sexual and reproductive health outcomes are associated with early menarche? Are such associations the same in low- and middle-income countries and high-income countries? What are some underlying factors, mediators, and moderators of these associations?

**Searches:**

Databases: We will search the following databases: PubMed, PsycINFO, Embase, CINAHL, POPLINE, ProQuest Social Science, Social Sciences Full Text, and Social Sciences Citation Index.

Search terms: A list of search terms identified through preliminary searches will be compiled, pilot tested, modified, and finalized to improve the effectiveness of the final search. The final searches will be conducted using both free text keywords and controlled vocabulary specific to each database to ensure that all relevant studies are captured.

**Types of study to be included:**

Observation studies including cohort studies, cross-sectional studies, case-control studies will be included. Case reports, case series and other studies with small sample sizes (i.e. <50 for quantitative studies; <15 for qualitative studies) will be excluded.

**Condition or domain being studied:**

Behaviors and experiences in adolescence or young adulthood pertaining to sexual and reproductive health (including early sexual debut, experiences of sexual advances from older men, early pregnancy and childbirth, sexual risk taking, unsafe sexual behaviors, unwanted pregnancy, sexual violence), sexually transmitted infections (STIs; including HIV) and early marriage.

We are not interested in other biological outcomes, genetic or hormonal factors, or antecedents of early menarche.

**Participants/population:**

Post-menarcheal females

**Interventions, exposures:**

Females who experienced early onset of menarche (Given that the age at menarche differs from one context to another, this will be assessed based on the definition used in each included study).

**Comparators/control:**

Females who experienced menarche at a regular age or those who experienced late menarche

**Context:**

Rural and urban settings in low- and middle-income countries

**Outcomes:**

Primary outcomes: Association between early menarche and sexual and reproductive health outcomes in adolescence and young adulthood including early sexual debut, experiences of sexual advances from older men, early pregnancy and childbirth, sexual risk taking, unsafe sexual behaviors, unwanted pregnancy, STIs and/or HIV, sexual violence, early marriage

Secondary outcomes: Underlying factors, mediators, and moderators of the associations between early menarche and the primary outcomes.

**Data extraction and coding:**

Studies identified through the database searches will be screened independently by two researchers using the following eligibility/exclusion criteria:

Eligibility criteria:

- Assesses the association between early menarche and at least one of the behavioral or experiential SRH outcomes of interest or sexually transmitted infection in adolescence or young adulthood (i.e. early sexual debut, experiences of sexual advances from older men, early pregnancy and/or childbirth, sexual risk-taking, unsafe sexual behaviors, unwanted pregnancy, STIs, HIV, sexual violence, early marriage)
- Peer-reviewed article
- Published in English
- Published since 1980

Exclusion criteria:

- Assesses the association between early menarche and biological factors (other than STIs) pertaining to SRH (e.g. genetic or hormonal aspects of early menarche, cancers related to early menarche, total fertility/fecundity)
- Assesses the antecedents of early menarche but not behaviors and experiences that occur after menarche (e.g. looks at the effects of father’s absence on age at menarche).

Disagreements will be resolved through discussion until consensus is reached. Once the final set of studies to be included in the review has been decided upon, two researchers will independently extract the following information from the included articles: Citation (1^st^ author and publication year), study design (type of study, sample size, participant characteristics, country of study, definitions/indicators used to measure outcomes and exposure), main findings (measures of association, effect sizes). Disagreements will be resolved through discussion until consensus is reached.

**Risk of bias assessment**

Risk of bias and quality assessment will be assessed concurrently with data extraction by two researchers independently using the Joanna Briggs Institute’s critical assessment review instruments for qualitative and quantitative studies. Based on the quality assessments, each study will be rated as strong, medium and weak. Weak studies will be excluded from the data synthesis.

**Strategy for data synthesis:**

We will conduct a thematic analysis of the findings from all included studies. Results from the analysis will be summarized and their programmatic and policy implications discussed.

**Analysis of subgroups or subsets**

High-income vs. low- and middle- income countries; rural vs. urban settings (as the data allows)

**Dissemination plans**

The results of the systematic review will be submitted for publication in a peer-review journal following the criteria listed in the Preferred Reporting Items for Systematic Reviews and Meta-Analysis (PRISMA) statement
